# Intricacy in the Diagnosis of Retroperitoneal Angiomyolipoma: A Rare Case

**DOI:** 10.7759/cureus.36357

**Published:** 2023-03-19

**Authors:** Bharati Thakur, K. M. Hiwale

**Affiliations:** 1 Pathology, Jawaharlal Nehru Medical College, Datta Meghe Institute of Higher Education and Research, Wardha, IND

**Keywords:** tumor, mesenchymal, extrarenal, angiomyolipoma, retroperitoneal

## Abstract

An uncommon benign mixed mesenchymal tumor called extrarenal retroperitoneal angiomyolipoma (ERAML) comprises thick-walled blood vessels, smooth muscle cells, and mature fat cells. Angiomyolipoma often develops in the kidneys, with extrarenal locations being uncommon. Many angiomyolipomas are unintentionally discovered when a person undergoes other medical issues. A 35-year-old man presented with a history of difficulty breathing and was on antibiotics. On follow-up, CT revealed a 22.5 × 14.3 × 8.6 cm right retroperitoneal mass. The retroperitoneal mass was resected en bloc at laparotomy and sent for histopathological examination. The grossly resected specimen was a smooth, well-circumscribed, yellowish-red retroperitoneal mass measuring 23 × 15 × 9 cm. The microscopic section reveals a tumor with prominent blood vessels and fatty tissue composed of spindle cells arranged into fascicles. The final diagnosis for this case is ERAML, which was challenging to distinguish from liposarcoma.

## Introduction

The three tissue components that make up an angiomyolipoma (AML) are mature fat cells, thick-walled blood vessels, and smooth muscle cells. AMLs were once thought to be hamartomas; however, it is now believed that they are perivascular epithelioid cell tumors (PEComas) [1]. Although extrarenal angiomyolipomas are very uncommon, they most usually affect the kidney. The most common extrarenal location is the liver. Less frequently, they occur in the pancreas, retroperitoneum, lung, breast, cardiac septum, and other areas of the gastrointestinal system [2]. Retroperitoneal tumors can result from various diseases, most commonly testicular malignancies, including lymphomas, liposarcomas, leiomyosarcomas, schwannomas, paragangliomas, neurofibromas, and other rare tumors [1]. On imaging investigations, patients with retroperitoneal AML typically present with spontaneous rupture, abdominal discomfort, or incidental abnormalities [3]. It may be difficult to distinguish between liposarcomas and extrarenal retroperitoneal angiomyolipomas (ERAMLs) before surgery as both include fatty components [4]. Here, we describe the case of a 35-year-old man with a right extrarenal retroperitoneal tumor measuring 23 × 15 × 9 cm that was discovered on a subsequent chest CT scan after receiving extensive treatment for shortness of breath.

## Case presentation

A 35-year-old man presented to the outpatient department of medicine with complaints of breathlessness, fatigue, lack of appetite, and stomachache and was on treatment with a long course of antibiotics. The blood picture showed microcytic anemia, and other biochemical investigations, including renal function, liver function, and electrolytes, were within the normal range on laboratory tests. An unremarkable chest CT was performed along with the abdomen and pelvis. A massive retroperitoneal mass was visible on CT abdomen scans, revealing an encapsulated mass measuring 22.5 × 14.3 × 8.6 cm in size. The right kidney was shifted ventrally and cranially. The patient was then referred to the surgery department for a surgical procedure. Exploratory laparotomy was done, and the retroperitoneal mass was resected en bloc and sent for histopathological examination. The right kidney was successfully saved and positioned in the standard retroperitoneal position.

**Figure 1:**
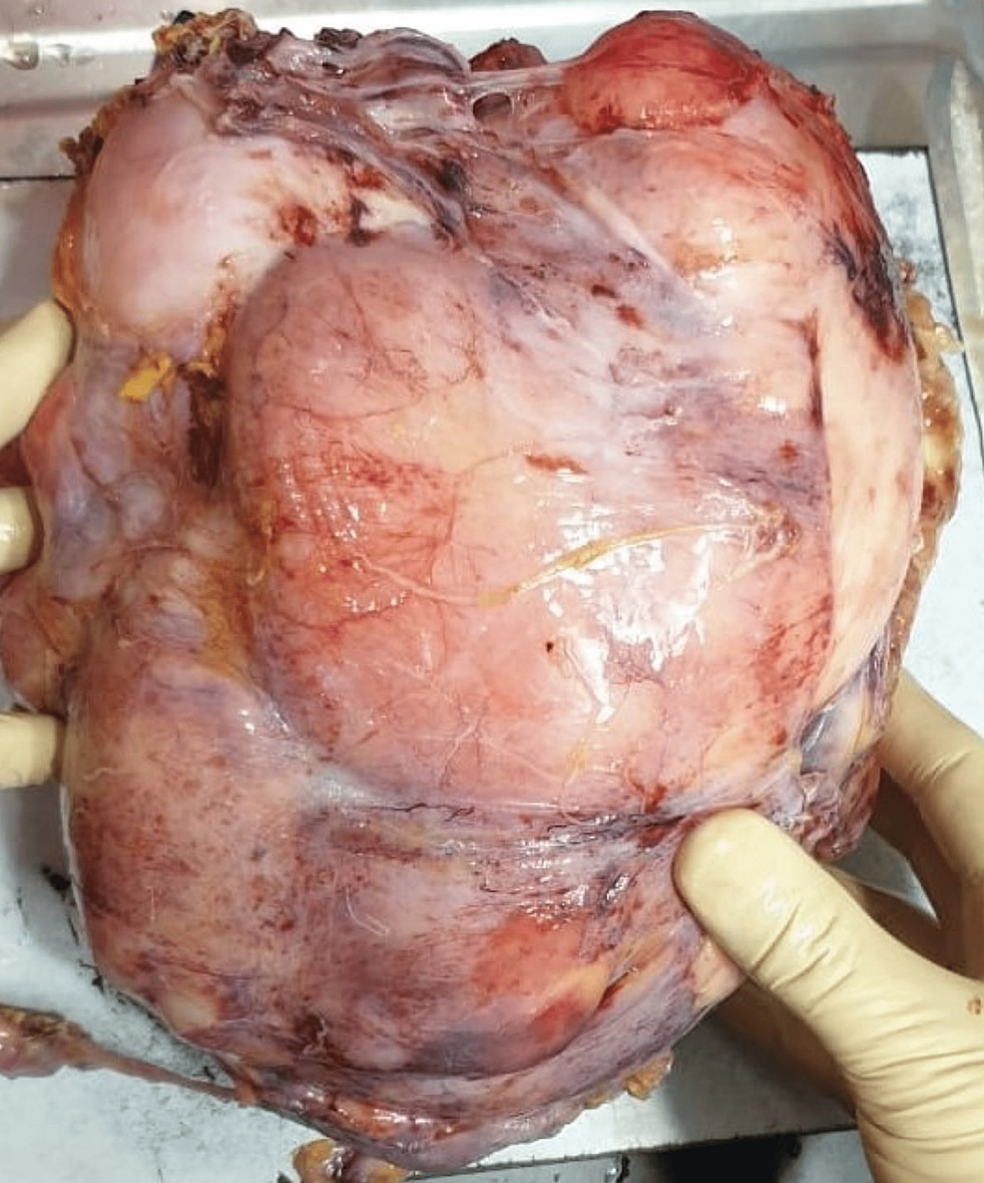
Gross resected specimen of the retroperitoneal mass.

**Figure 2:**
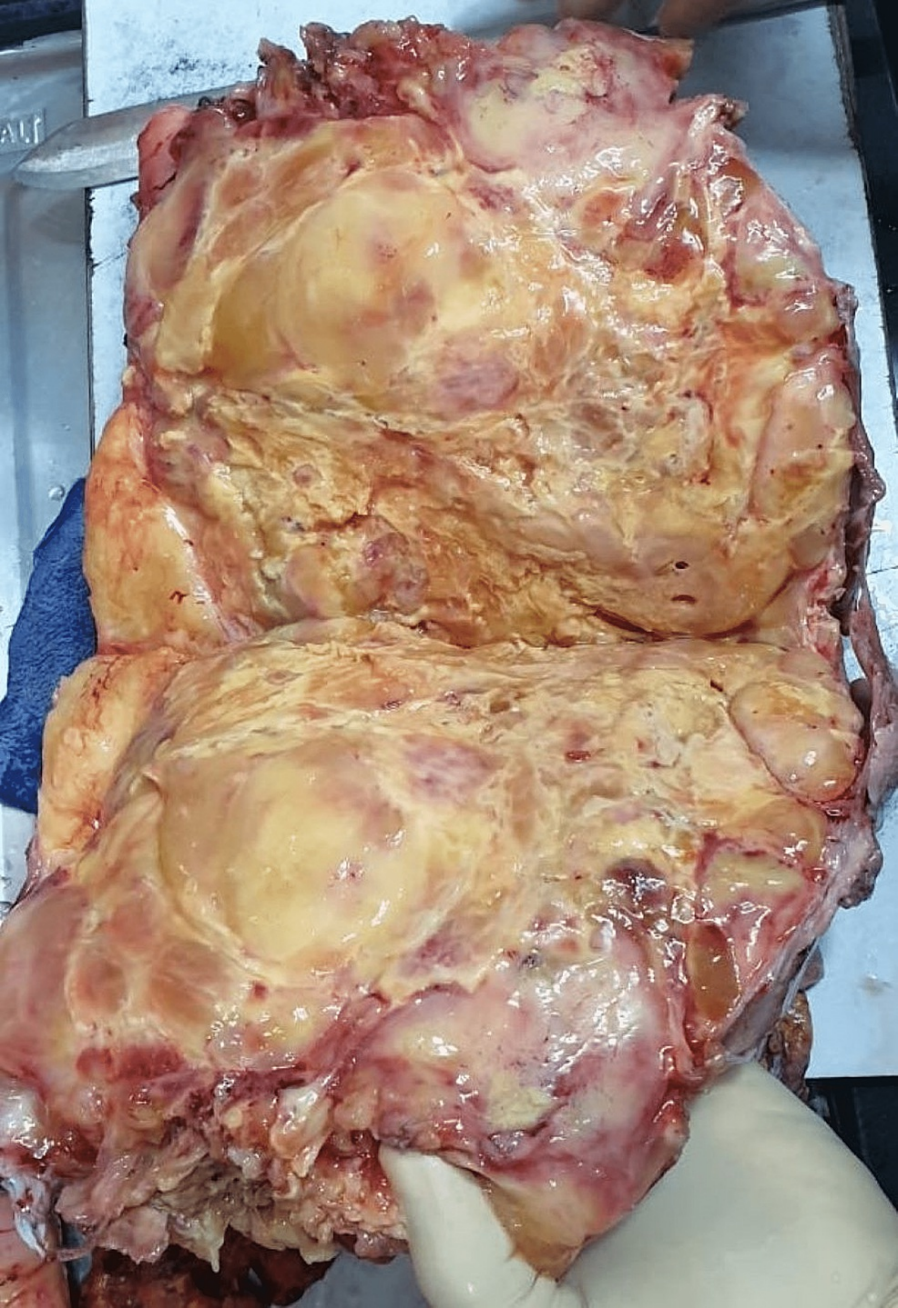
Cut section showing the yellowish-red area.

**Figure 3:**
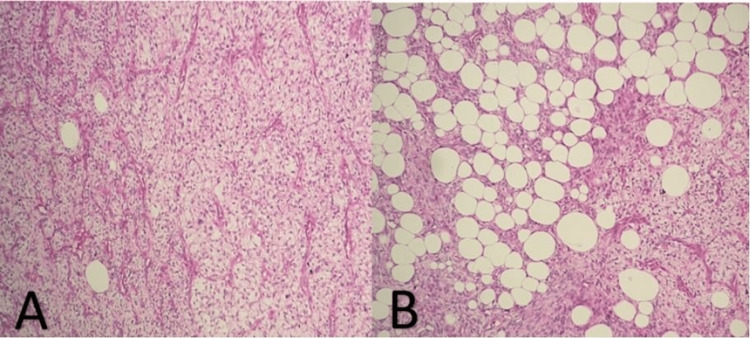
(A & B): Hematoxylin and eosin section (10×) showing a combination of the three components, including smooth muscle cells, mature fat cells, and thick-walled blood vessels.

The resected retroperitoneal mass was grossly smooth, well-circumscribed, and yellowish-red in color, measuring 23 × 15 × 9 cm in size (Figure 1). On the cut, the mass showed a yellowish-white heterogeneous area with a few hemorrhagic foci (Figure 2). Histopathology revealed characteristics of a confined tumor comprising adipose tissue and muscle-like tissue with large muscular blood vessels (Figures 3A, 3B). There was no significant nuclear atypia. Mitotic activity was not apparent. A final histopathological diagnosis of angiomyolipoma was made because of the characteristic histomorphological features.

## Discussion

Fischer first identified the case of angiomyolipoma in 1911, and, in 1951, Morgan used the word angiomyolipoma. Initially, angiomyolipoma was described as choristoma or hamartoma [5]. Angiomyolipomas are uncommon, complex mesenchymal tumors that typically grow in the kidney and comprise thick-walled blood vessels, mature adipose tissue, and smooth muscle cells. When patients are scanned for other ailments, a rare tumor known as ERAML usually manifests as an incidentaloma. AMLs, which comprise around 1% of all renal masses, are frequent primary renal parenchymal tumors, whereas ERAMLs are extremely rare [6]. AMLs are unusual mesenchymal tumors that typically develop from the renal parenchyma; however, they can also develop from other locations such as the oral cavity, colon, liver, nasal cavity, lung, adrenal glands, skin, and bladder [5].

Due to their ability to mimic benign and malignant retroperitoneal tumors, retroperitoneal ERAMLs pose a diagnostic and therapeutic challenge [6]. The differential diagnoses for the lesion’s location include renal cell cancer, retroperitoneal liposarcoma, lipoma, AML, adrenal adenocarcinoma, and leiomyoma with fatty alteration [6]. Yet, despite their rarity and surgical pathologists’ lack of acquaintance with them, AMLs and leiomyomas with fatty change must be separated from liposarcomas because they are benign [4].

AMLs can be difficult to accurately diagnose at an extrarenal location because of their varying imaging appearances across various imaging modalities. However, AMLs are likely detected in preoperative radiology. Intratumoral fat and central vessels have been seen, which support the diagnosis. However, in our case and the others, the accurate diagnosis was not made until after a laparotomy [7].

The two imaging modalities most frequently used to study AMLs are CT and CT angiography. To differentiate ERAMLs from liposarcomas, Wang et al. examined the abdominal CT scan radiological features depicting ERAMLs. Using MRI, perinephric and retroperitoneal AMLs can be treated while defining the anatomical link between ERAMLs, the kidney, and its vasculature. Along with CT imaging, MRI can also be used [6].

The most frequent form of treatment for ERAML is surgery. The surgeon must perform a frozen histological section to protect the renal system if the tumor is close to the kidneys. Selective artery angiography and embolization have been used successfully to reduce bleeding in patients in whom retroperitoneal hemorrhage constitutes an emergency, typically enabling elective surgical removal [8].

Histopathologically, AML has three components, namely, myoid component, vascular component, and adipose tissue component in variable proportion. This unique histopathological appearance of AML helps rule out other differential diagnoses such as lipomatosis, lipoma, leiomyoma, lipoleiomyoma, and liposarcoma. An immunohistochemistry analysis can be performed to confirm the diagnosis. Immunohistochemically AML is positive for smooth muscle actin, HMB-45, and Melan A. The vascular component is positive for CD 34, and S-100 immunopositivity is seen in the adipose tissue component [9].

AML can be correctly diagnosed to avoid unnecessary surgery because liposarcoma can only be treated surgically, whereas AML can be treated with persistent surveillance and conservative medical care [4].

## Conclusions

The pathologist must identify this lesion even if seen in the retroperitoneum outside of the kidney because it may mimic other retroperitoneal benign and malignant tumors that need to be distinguished. In addition to highlighting the challenge in diagnosing retroperitoneal malignancies, our case also provides information on another instance of retroperitoneal AML. Hence, it is necessary to use imaging and histologic correlation. A proper diagnosis is crucial for the patient’s ongoing care and to prevent unnecessary treatments.
